# A timely update on g-C_3_N_4_-based photocatalysts towards the remediation of Cr(vi) in aqueous streams

**DOI:** 10.1039/d4ra07350a

**Published:** 2024-11-18

**Authors:** Sambhu Prasad Pattnaik, Upali Aparajita Mohanty, Kulamani Parida

**Affiliations:** a Centre for Nano Science and Nano Technology, Institute of Technical Education and Research, Siksha ‘O’ Anusandhan (Deemed to be University) Bhubaneswar 751030 India kulamaniparida@soa.ac.in sp1pattnaik@gmail.com +919776645909 +919437647766

## Abstract

Hexavalent chromium (Cr(vi)) is a prominent carcinogen. In environmental engineering, the elimination of hexavalent chromium from aqueous media is a noteworthy field of study. In this regard, nanoparticle science and technology have contributed significantly to the photocatalytic reduction of Cr(vi). In this review, a methodical search was undertaken to discover the most recent advancements in the field of photocatalytic reduction of Cr(vi) utilizing g-C_3_N_4_ and composites derived from it. This paper deals with the advancements and applications of g-C_3_N_4_ and its composites in the Cr(vi) remediation of water-borne pollutants. Different intriguing systems, suggested by various researcher groups, have been discussed. Different characterization techniques often conducted on photocatalysts based on g-C_3_N_4_ have also been highlighted so as to gain an understanding of the Cr(vi) removal process. Lastly, the future scope of the g-C_3_N_4_-derived photocatalysts, present challenges, and the viability of employing these photocatalysts in an extensive treatment plant have been discussed.

## Abstract 2

Photocatalytic Cr(vi) reduction using g-C_3_N_4_.

## Graphical abstract

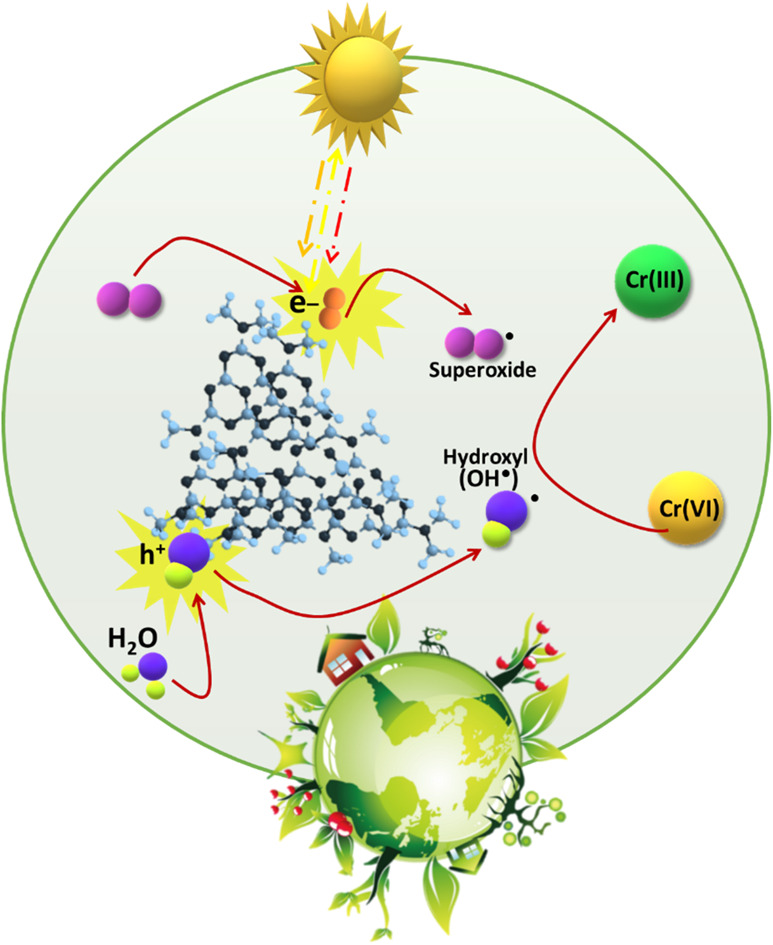

## Introduction

**Fig. 1:**
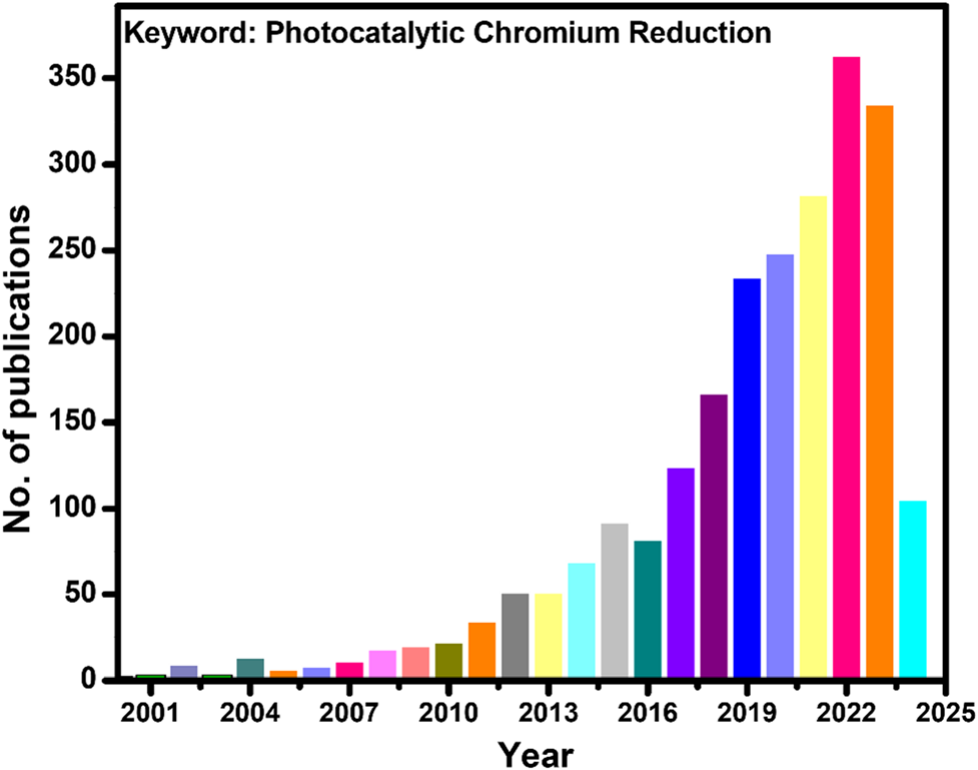
Schematic showing the number of published articles on photocatalytic chromium(vi) reduction in the last twenty-four years.

Aquatic heavy metal pollution in recent decades has remained challenging for the industrialized world. Effluents from steel production, electroplating and leather tanning industries carrying chromium-containing wastewater find their way into the water bodies. Among the various existing oxidation states of chromium, hexavalent chromium is the most toxic and well-known for its carcinogenicity, teratogenicity, and mutagenicity.^1–3^ In the list of various toxic substances compiled by the ATSDR (Agency for Toxic Substances and Disease Registry) of the United States Department of Health and Human Services, Cr(vi) occupies a significant place at 17th.^4^ If Cr(vi) comes in contact with blood, it has the potential to form a stable Cr-hemoglobin complex and can stay in the bloodstream for about 120 days,^5,6^ impacting health. Because of its toxicity, the threshold value of Cr(vi) concentration in wastewater prior to discharge outside the plant boundary is fixed below 0.05 mg L^−1^ by regulatory authorities in many countries.^7^ This statutory requirement necessitates the treatment of wastewater until the limit of Cr(vi) concentration is maintained below 0.05 mg L^−1^ before releasing it to the water bodies. Cr(vi) is not biodegradable, and therefore, the necessity for its degradation is quite thought-provoking from an environmental point of view. Various methods have been investigated regarding the eradication of Cr(vi) present in wastewater, with the preferred method being adsorption. However, the ability of various adsorbents is hindered by their limited adsorption and many more challenges, like the cost associated with chemicals and release of secondary pollutants. Currently, there is a shift of attention towards an eco-friendly method for reducing hexavalent chromium *via* the utilization of semiconducting photocatalysts. The graph presented in Fig. 1 displays the number of research papers published in recent years in the arena of photocatalytic hexavalent chromium reductions.

**Fig. 2:**
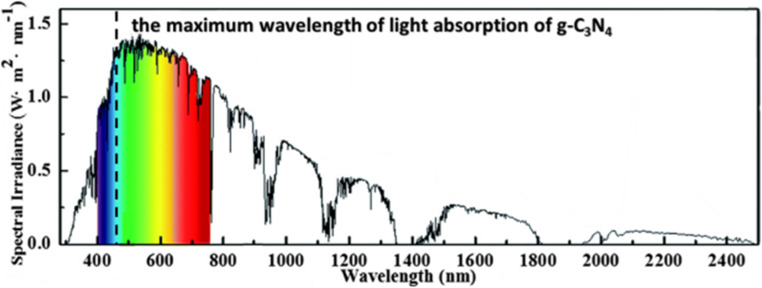
Schematic exhibiting the maximum wavelength of light absorption of g-C_3_N_4_. Reproduced with permission from ref. 14, Copyright 2023, Elsevier.

Photocatalysts that convert renewable solar energy into chemical energy are widely used in CO_2_ reduction, H_2_ generation, nitrogen fixation, chromium reduction,^8–12^ organic compound degradation and sterilization of potable water.^13^ Cr(vi) reduction through photocatalysis has garnered profound attention as photocatalysis employs sunlight as the energy source and abundant H_2_O and O_2_ as raw materials without generating any harmful by-products. Prior studies have extensively demonstrated profuse photocatalytic semiconductor materials in terms of Cr(vi) reductions such as (TiO_2_, g-C_3_N_4_ and other photocatalysts). Among the extensively studied and popular photocatalysts towards Cr(vi) remediation, it is found that while TiO_2_ offers more energetic UV-driven photocatalysis along with high catalyst surface area, it suffers from demerits like wide bandgaps, low absorption of visible-light spectrum and significant charge carrier recombination. Similarly, another photocatalyst that works well with both UV and visible radiation is ZnO, with the limitation of charge carrier recombination and surface defects affecting performance. Ag_3_PO_4_ exhibits high photocatalytic efficiency but is prone to photo corrosion and impaired by its limited visible light absorption. Bi_2_WO_6_ shows good activity for Cr(vi) reduction but has issues like low visible light absorption and possible reactive catalyst surfaces. CdS, as an effective visible-light photocatalyst, demonstrated low visible-light absorption, higher electron–hole recombination and toxicity concerns. On the other hand, versatile g-C_3_N_4_ demonstrated unique selectivity, visible-light absorption and no toxicity issues. Some demerits remain with the pristine material, like low specific surface area, low quantum efficiency and charge carrier recombination, all of which could be taken care of with appropriate strategies like compositing with other materials. Compared to other photocatalysts, g-C_3_N_4_ has merits in terms of photon wavelengths of approximately 460 nm conforming to its bandgap energy, making it well matched to visible light response, well represented in Fig. 2.

In addition, its grid structure containing heptazine and *s*-triazine rings with delocalised π electrons is favourable for electron and hole transport. g-C_3_N_4_, being an organic semiconductor, has room for easy modification and alteration of energy levels by incorporating heteroatoms. Since the discovery of g-C_3_N_4_'s capability to catalyse water decomposition in 2009 by Wang and coworkers,^15^ it has grabbed wide attention for its excellent photocatalytic efficiencies. As a result, g-C_3_N_4_-derived composites have found substantial application in photocatalysis, including chromium(vi) reduction.

**Fig. 3:**
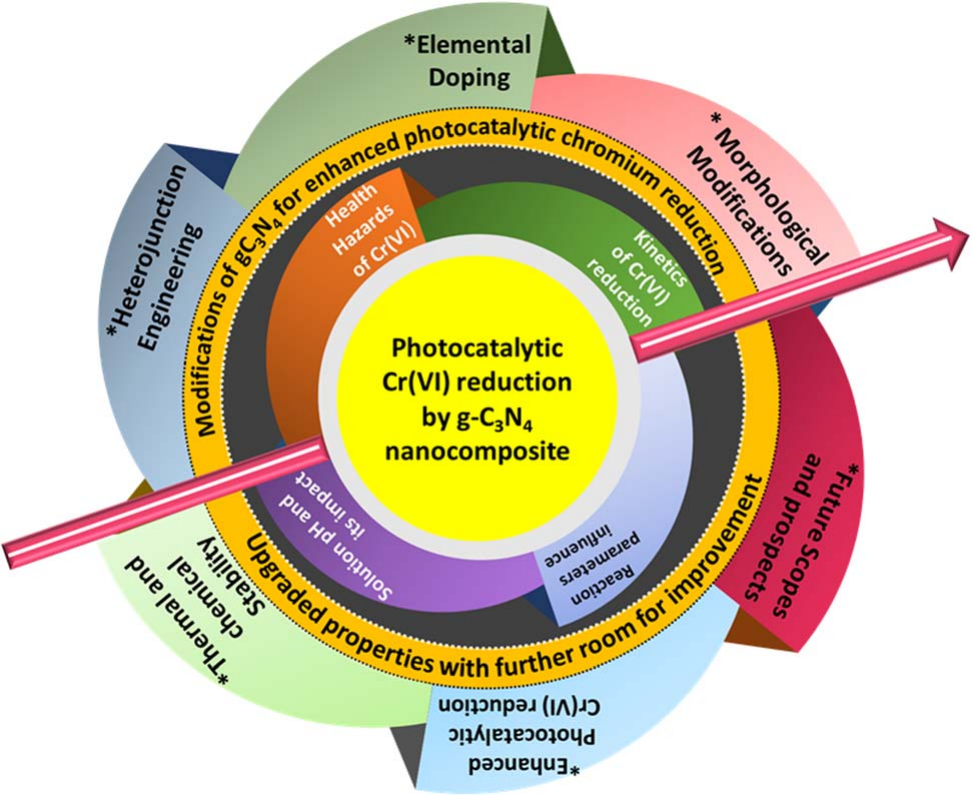
Schematic illustration of the content of the review article.

So far as Cr(vi) reduction in aqueous media by photocatalysis is concerned, there exists space for improvement in targeted chromium reduction efficiency essential for real-world applications, and consistent efforts need to be dedicated to the modification and development of PC. As the assessment of g-C_3_N_4_ continues to advance in a fast-paced manner, inclusive reviews and surveys are vital for staying abreast of the newest improvements across various aspects of this material, indispensable for the future. In favour of this, there is a demand for a timely and comprehensive review concerning the latest advances of g-C_3_N_4_ functionalization strategies towards photocatalytic reduction of Cr(vi). Herein, a run-through on g-C_3_N_4_ modified photocatalytic nano-heterostructures for the photocatalytic Cr(vi) reduction is well served. The review presents insights into the potential toxicity of Cr(vi) invasion in wastewater and follows it with the impressive photocatalytic properties of g-C_3_N_4_ and various modifications for dealing with critical edge chromium(vi) reduction. Furthermore, this review intends to provide the know-how in the area of g-C_3_N_4_ modification strategies and motivate the researchers to attempt new alteration routes for g-C_3_N_4_ for efficient photoreduction of Cr(vi). The schematic representation of the content of the review is presented in Fig. 3.

## Principle of photocatalytic Cr(vi) reduction

### Exposure to humans and health issues linked to Cr(vi)

Hexavalent chromium has been considered to be in a heightened toxic oxidation state compared to its trivalent counterpart, owing primarily to the excellent capability of easy cellular membrane permeability.^16^ In this case, two oxyanions are predominantly formed in water in the presence of protons, CrO_4_^2−^ and CrO_7_^2−^, which undergo reversible reactions [eqn (1)].

1 2CrO_4_^2−^ + 2H_3_O^+^ ↔ CrO_7_^2−^ + 3H_2_O

Cr(vi) seamlessly undergoes reduction to the Cr(iii) state just after entering the cell membrane, which leads to complex formation with intracellular macromolecules, including genetic materials.^17^ Cr(vi) easily permeates through the cell membranes by forming free radicals, which cause DNA alterations and can subsequently lead to birth defects and reduced reproductive health (Chen *et al.*, 2019).^18^ The intracellular Cr(vi) reduction can interact with DNA, producing genotoxic effects, altered immunological responses, and interrupted signalling pathways.^19^ Additionally, Cr(vi) is recognised as a deadly carcinogen and teratogen causing skin abnormalities.^20^ Potential threats to plants are also caused by the presence of Cr(vi), which interferes in the process of photosynthesis by decreasing nutrient uptake.^21^ Various biochemical processes are also affected, generating reactive oxygen species in plant tissues and causing toxicity like chlorosis and necrosis.^22^

### Kinetics of the Cr(vi) reduction process

Investigating the kinetics governing the reaction mechanism during the degradation of contaminants helps in our understanding of pollution abetment. The photocatalytic Cr(vi) reduction reaction invariably starts with the adsorption of Cr(vi) on the photocatalyst (PC) surface, after which it undergoes photoreduction. In order to comprehend the photocatalytic efficiency of Cr(vi) reduction, the frequently employed method is the Langmuir Hinshelwood pseudo 1st order model. The adsorption rate as a function of different factors is presented in [eqn (2)].2
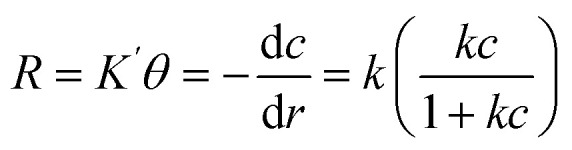
Here, *R* represents the rate of reduction, *k* represents the reduction rate constant, *c* represents the concentration of the contaminants, 
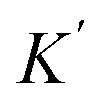
 is the adsorption coefficient and *θ* represents the reactant site coverage. Additionally, for the lower concentration solution, the L–H model follows pseudo 1st order rate kinetics by eqn (3) and (4).3
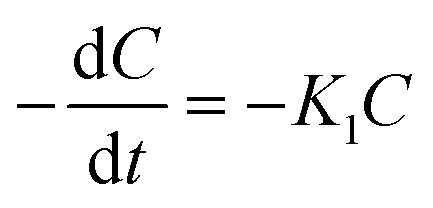
4
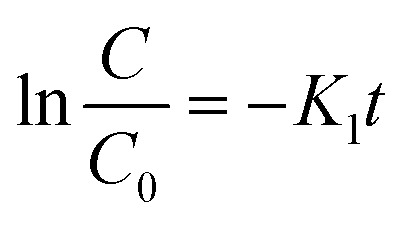


*K*_1_ is the pseudo-first-order rate constant, *t* represents the time of reaction, and *C* and *C*_0_ are the final and initial concentrations of the contaminant, in that order.

When light falls on a semiconducting material, it absorbs light and if the energy of the absorbed photon is more than the semiconductor bandgap, then the photocatalytic mechanism is initiated. Consequently, electrons in the semiconductor's valence band get excited to its conduction band, creating holes in the valence band. The photocatalytic reduction of Cr(vi) is caused by photoinduced electrons on the conduction band (C_B_) of the semiconductor. The feasibility of Cr(vi) reduction is dictated by a condition that the conduction band edge has to be at a higher negative potential than the Cr(vi)/Cr(iii)/s redox potential. This requirement is normally fulfilled by the chosen semiconductor. As Cr(iii)/Cr(0) demonstrates a higher negative redox potential, the reduction of Cr(vi) to the metallic state is not possible, *i.e.*, (Cr(0)) by photocatalysis. The photogenerated electrons may move directly to the adsorbed Cr(vi) or to the photocatalyst's surface boundary. The movement of electrons from C_B_ to Cr(vi) can happen in two possible ways.^23,24^ It is possible that Cr(vi) could be directly reduced to Cr(iii) by a single-step transfer involving three electrons from the C_B_ of the photocatalyst as per eqn (5). This mechanism appears to be more probable when there are hole scavenger molecules available in the medium, enhancing the charge separation of electrons and holes and leading to more electrons being available for the reaction.^25^ On the other hand, Cr(vi) is photoreduced through three sequential single electron transfers and obviously a very slow process as per eqn (6), producing intermediates of Cr(v) and Cr(iv).^26,27^ As seen in eqn (5), the photoreduction of Cr(vi) utilizes H^+^ ions, preferring an acidic medium. The possibility of unwanted reverse reactions, *i.e.*, Cr(iii) oxidizing to Cr(vi) by oxidative positive holes and reactive oxidative species (ROS) on the surface of the photocatalyst, cannot be ruled out as per eqn (7). It is always preferred to add hole scavenger molecules in the aqueous medium so that they can react with oxidative species like positive holes and ROSs.^28^ The effectiveness of the photocatalytic reduction process depends on the properties of hole scavenger molecules used.^29,30^

5 Cr_2_O_7_^2−^ + 14H^+^ + 6e^−^ → 2Cr^3+^ + 7H_2_O

6 Cr(vi) + e^−^ → Cr(v) + e^−^ → Cr(iv) + e^−^ → Cr(iii)

7 Cr(iii) + ROSs → Cr(vi)

8 O_2_ + e^−^ → *O_2_^−^

9 *O_2_^−^ + Cr(vi) → Cr(v) + O_2_

10 HCrO_4_^−^ + 7H^+^ + 3e^−^ = Cr^3+^ + 4H_2_O

11 CrO_4_^2−^ + 4H_2_O + 3e^−^ = Cr(OH)_3_ + 5OH^−^

**Fig. 4:**
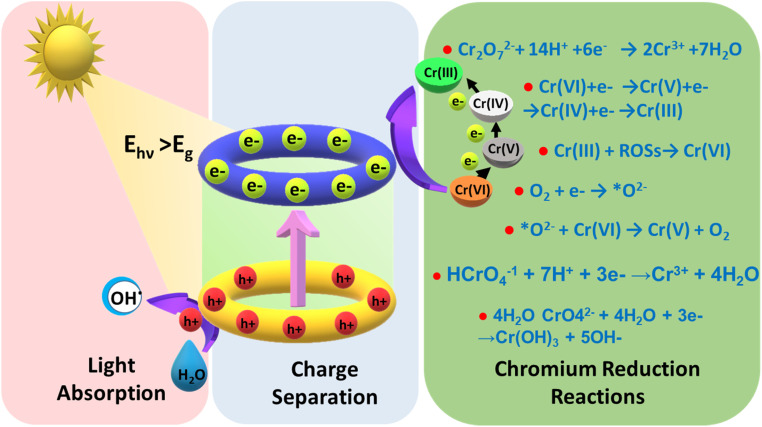
Schematic showing the photochemical chromium(vi) reduction process.

In a pure aqueous medium, free of hole scavenger molecules, the photogenerated *O_2_^−^ on the C_B_ can go for the reduction of Cr(vi) to Cr(v) in eqn (8)–(11).^31,32^ However, in real-life situations, chrome-laden aqueous streams do contain organic contaminants that work as hole scavengers. A pictorial illustration of the photocatalytic reduction of hexavalent chromium procedures using semiconducting photocatalysis is displayed in Fig. 4.

### Influence of reaction parameters

Factors influencing Cr(vi) photocatalytic reduction include types of light sources and intensities thereof, presence of hole scavengers, initial Cr(vi) concentration, pH of the medium and catalyst dosage. The source of light governs the light intensity and frequencies of emitted radiations, *i.e.*, the quality and quantities of photons released. The photon should be capable of knocking off electrons in the V_B_ of the semiconductor PC to its conduction band. The more energetic the photon, the better it is. Photons with lesser energy than PC's band gap are of no use. Similarly, a higher number of useful photons from intense beams commits a higher number of photoinduced electrons for participation in photocatalysis.

While electrons in the C_B_ participated in photocatalysis to provide meaningful reaction products, at the same time, holes, a highly oxidising species in the V_B_, remain in the absence of a scavenger. In order to drive the reaction forward unhindered, the holes need to be consumed in the overall reaction or scavenged out by using a hole scavenger, failing which will hinder the overall photocatalytic process. Thus, we can see that the hole scavenger impacts the photocatalytic process considerably. The initial Cr(vi) concentration denoted by *C*_0_ is a critical parameter. The photocatalytic reaction efficiency depends on it when other conditions remain unaltered. At lower *C*_0_, the PC effect is more pronounced. The solution pH is a crucial factor for Cr(vi) reduction as the reaction is facilitated by lower pH. The PC concentration impacts the photocatalytic reaction. For a given photocatalyst, say in this case, g-C_3_N_4_ composite and initial chromium concentration of *C*_0,_ the catalyst dose requires to be optimised for a given light source of particular intensity as more catalyst dosage beyond a certain limit (to be determined experimentally) may cause decrease in the photocatalytic degradation rate due to increase in the turbidity of the reaction medium and consequent decrease in light intensity in the reaction medium, which in turn causes lower light absorption by the photocatalyst.

The presence of organic materials in rejected water streams plays a crucial role in the photocatalytic degradation of Cr(vi). In fact, the Cr(vi) bearing wastewater streams, in reality, contain organic matter as well. At times, these materials are oxidized on the catalyst surface, consuming photogenerated holes and acting as a scavenger for holes that help boost the photocatalytic degradation of Cr(vi). That is why many research publications describe Cr(vi) photocatalytic reduction and simultaneous oxidation of the organic matter together.

### Solution pH and its impact

The pH of the aqueous medium is a very important aspect in deciding the rate of the photocatalytic reduction of Cr(vi) as H^+^ ions participate in the overall reaction process. Due to electrostatic interactions, the protons also help pull the photoinduced electrons from the C_B_ of PC to the media containing Cr(vi). Hence, an acidic medium always favours the photocatalytic reduction of Cr(vi) and is almost negligible at an alkaline pH.^29,33^

## Physicochemical propertied of g-C_3_N_4_

Over the years, g-C_3_N_4_, owing to its 2-D π conjugated polymeric, narrow band gap, and metal-free semiconductor characteristics, has emerged as an eye-catching material for various applications in the fields of sensors, supercapacitors, adsorptions and photocatalytic redox reactions. The physicochemical characteristics of g-C_3_N_4_ play a pivotal in the photocatalysis avenue, while another essential property of g-C_3_N_4_ is stability. g-C_3_N_4_, with significant thermal stability, allows this material to be utilized in high-temperature reactions. In addition to its thermal stability up to 600 °C, g-C_3_N_4_ also possesses good chemical stability across a varied pH range, *i.e.*, immune to acids and alkalis.^34,35^ The higher negative displacements of zeta potential provide better stability in aqueous solutions debarring agglomerations. The easy-to-exfoliate layered structure of g-C_3_N_4_, along with various functional amino and imino groups, enhances its dispersibility, providing enhanced active sites for interactions during the reactions. Among a plethora of sustainable next-gen photocatalysts, nanostructured g-C_3_N_4_ occupies a prominent place. This polymeric, metal-free photocatalyst has a graphitic planar structure consisting of conjugated tris-*s*-triazine rings in abundance. Its structure provides sufficient π bonded electrons excitable on absorbing visible radiation. Due to this, it exhibits sensitivity to the visible range of solar light thanks to its visible light-driven band gap and appropriate band edges.^36–38^ g-C_3_N_4_ is easy to prepare at low cost from precursors rich in earth-abundant materials like nitrogen and carbon. It can be prepared from precursors rich in contents of carbon and nitrogen, such as urea,^39–44^ melamine^45–55^, cyanamide,^56–59^ dicyandiamide,^60–63^ ammonium thiocyanate,^64^ and thiourea.^65–67^ As the band gap of g-C_3_N_4_ is 2.7 eV, it can effectively absorb visible radiation of less than 450 nm wavelength, implying future prospects in solar energy utilization applications. Besides these, it is nontoxic in nature eliminating the need of handling issues. The photocatalyst is easy to separate and amenable to recycle and reuse.

**Fig. 5:**
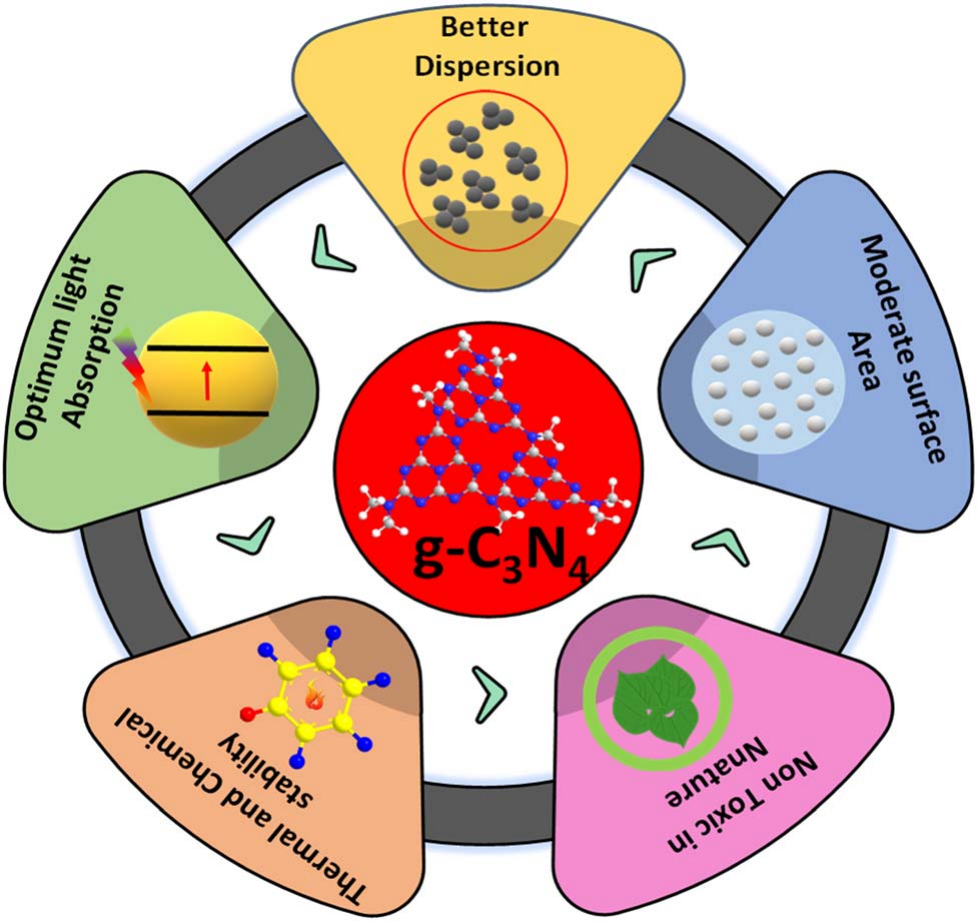
Schematic displaying the properties of g-C_3_N_4_.

In spite of the advantages of g-C_3_N_4_ as a photocatalyst, certain inherent bottlenecks^68^ hinder the performance of pristine g-C_3_N_4_ towards practical use to date. This is partly because standard synthesis methods of thermal poly-condensation of precursors produce relatively thick materials consisting of stacked *s*-triazine layers. The stacking of *s*-triazine layers creates bulk g-C_3_N_4_ materials with low specific surface areas. Lower SSA leads to fewer available reaction sites, impeding the photocatalytic reaction kinetics. Other bottlenecks in the photocatalytic activity of g-C_3_N_4_ include different factors, such as fast photogenerated charge carrier recombination,^69^ poor crystallinity and surface defects. Researchers, to this day, have been working on optimizing synthesis techniques of g-C_3_N_4_ to modify its physicochemical properties and photocatalytic efficiency. The design and fabrication of g-C_3_N_4_ composites of suitable band structure could improve the charge separation efficacy, thereby enhancing the photocatalytic performance. Hence, the majority of work in this field of research is devoted to the construction of suitable g-C_3_N_4_ derived composites with suitable band structure, increased porosity and surface area, enhancing its overall photocatalytic performance. A summarized representation of all the properties of g-C_3_N_4_ is given in Fig. 5.

Exfoliation of g-C_3_N_4_ is carried out to obtain nanosheets, which improve the SSA. The high energy conversion efficiency of g-C_3_N_4_ as a photocatalyst may be achieved by stimulating the efficient parting and migration of photoinduced charge carriers, expanding the spectral response range and increasing its specific surface area. All of these shortcomings of g-C_3_N_4_ could be alleviated to some extent by doping foreign materials into the g-C_3_N_4_ matrix using copolymerization and other techniques to change the electronic and band arrangement of g-C_3_N_4_. The heterojunctions made on g-C_3_N_4_ are an outcome of effective strategies^70,71^ to design and fabricate amalgamated photocatalysts. The g-C_3_N_4_ heterostructures distinctly enhance the photocatalytic performance of g-C_3_N_4_ by improving the parting and transfer of photoinduced charges, broadening the light absorption range, and widening redox potentials, attributable to the existence of both built-in electric fields at the g-C_3_N_4_ interface and other material-making composites and the complementarity between the g-C_3_N_4_ electronic structure and that of the constituent material. Doping with impurities such as metals, nonmetals or nanoparticles also helps lower the recombination efficiency of photoinduced e^−^–h^+^ pairs.

## Modification of g-C_3_N_4_ for promoting Cr(vi) reduction

### Heterojunction formation

The g-C_3_N_4_ heterojunction catalyst is synthesized by combining g-C_3_N_4_ layers with layers of other materials like semiconductors so that an interface between the two layers may be created. The g-C_3_N_4_ heterojunction is always beneficial compared to pristine g-C_3_N_4_ in photocatalytic applications. The heterojunction reduces the recombination of charge carriers on the interface and thereby improves the photocatalytic activity of a PC.

**Fig. 6:**
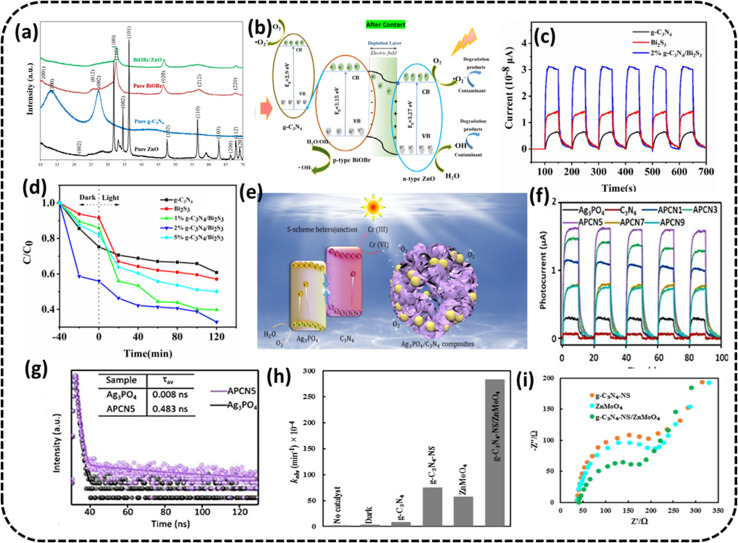
(a) XRD analysis of ZnO, BiOBr, BiOBr/ZnO and g-C_3_N_4_ samples. (b) Schematic depicting the energy band structure of PCs after the formation of the ZnO/BiOBr heterojunction and probable mechanism of photocatalysis. Reproduced with permission from ref. 72 Copyright 2023, Elsevier. (c) Photocurrent response of pristine g-C_3_N_4_, Bi_2_S_3_, and 2% g-C_3_N_4_/Bi_2_S_3_ (d) photocatalytic efficacy of Bi_2_S_3_, g-C_3_N_4_, and 2% g-C_3_N_4_/Bi_2_S_3_: reduction of Cr(vi) exposed to visible radiation. Reproduced with permission from ref. 73, Copyright 2022, Avanti Publishers. (e) Schematic of the Ag_3_PO_4_/C_3_N_4_ composite with S-scheme heterojunction (f) transient photocurrent responses (g) time-resolved PL decay spectra reproduced with permission from ref. 74 Copyright 2022, FJIRSM, CAS, Fuzhou (h) Cr(vi) reduction rate over the prepared photocatalysts, (i) EIS curves for the fabricated photocatalysts Reproduced with permission from ref. 75 Copyright 2022, Springer Nature.

Nguyen *et al.* reported^72^ that the g-C_3_N_4_ and n–p type ZnO/BiOBr heterojunction resembling a flower was synthesized using a hydrothermal method, with the potential for Cr(vi) removal in aqueous media. They determined the phase structures and catalyst purity from the XRD patterns of pristine ZnO, pristine BiOBr, pristine g-C_3_N_4_, and the BiOBr/ZnO heterojunction, as shown in Fig. 6a. The optimal PC with catalyst amounts of 0.05 g g-C_3_N_4_ and 0.4 g ZnO/BiOBr-2 (molar ratio Bi/Zn = 2) were seen to accomplish 96% of Cr(vi) elimination in 180 min with measured rate constant = 0.0105 min^−1^ under simulated UV light. The experiments were conducted with a *C*_0_ = 10 mg L^−1^ of Cr(vi) with a pH value of 2. The PC could be reused without loss of efficiency for four consecutive cycles, indicating its good photocatalytic stability. The improvement in performance was attributed to fewer recombining charge carriers at the boundary and a rise in the range of absorption of UV light. The well-presented schematic illustration of the reaction mechanism is presented in Fig. 6b.

Ding *et al.* synthesized Bi_2_S_3_@g-C_3_N_4_ Z-scheme heterojunctions^73^ using the hydrothermal method. The optimal PC of 2% g-C_3_N_4_/Bi_2_S_3_ could reduce 10 mg L^−1^ Cr(vi) solution to the tune of 93.4% in 120 min under simulated solar radiation, as displayed in Fig. 6c. The improved photocatalytic activity of PC was ascribed to a better segregation and movement of the charge carriers, and its optimum band structure helped increase the range of light absorption.

The current trend of PC exposed to visible light reveals the separation efficiency of photoinduced charge carriers. The photocurrent responses of pristine g-C_3_N_4_, Bi_2_S_3_, and 2% g-C_3_N_4_/Bi_2_S_3_ are shown in Fig. 6d, where the photocurrent rises as mentioned: pristine g-C_3_N_4_ < Bi_2_S_3_ < 2% g-C_3_N_4_/Bi_2_S_3_, of which the photocurrent of optimal PC 2% g-C_3_N_4_/Bi_2_S_3_ is considerably more than that of pure g-C_3_N_4_ and Bi_2_S_3_. It was shown that the composite of g-C_3_N_4_ and Bi_2_S_3_ radically improved the separation rate of photoinduced charge carriers, leading to the enhancement of the photocatalytic redox rate of g-C_3_N_4_/Bi_2_S_3_ composites.

Yang *et al.* created an Ag_3_PO_4_/g-C_3_N_4_ heterojunction composite^74^ by coupling Ag_3_PO_4_ particles with g-C_3_N_4_ hollow spheres *via* an *in situ* precipitation method. The S-scheme heterojunction amidst Ag_3_PO_4_ and g-C_3_N_4_ could hasten the charge segregation and improve the photoreduction ability, as can be predicted from the transient photo current response shown in Fig. 6e. The highest transient photocurrent response came from optimized PC APCN5, implying the best expected photocatalytic response, as displayed in Fig. 6e. Time-resolved PL decay spectra in Fig. 6f also corroborated the expectation from PC as the extended fluorescence lifetime of APCN5 implied boosted disassociation of photogenerated e^−^–h^+^ pairs. The said g-C_3_N_4_ hollow sphere structure could accommodate a higher number of active sites in the photocatalytic process, resulting in an 87.9% reduction of a 20 mg L^−1^ initial Cr(vi) solution in 75 min under visible light using the optimized composite denoted as APCN5 having 5% carbon spheres.

Mousavi *et al.* fabricated a heterojunction^75^ in which ZnMoO_4_ was loaded on g-C_3_N_4_ nanosheets by a calcining-hydrothermal method and the resultant composite was shown to reduce Cr(vi) in 120 min with the rate constant of 284 min^−1^, as presented in Fig. 6g. The EIS measurements revealed the separation efficiency and interfacial charge transfer resistance of PCs (Fig. 6h). The Nyquist plot of g-C_3_N_4_-NS/ZnMoO_4_ possessed the smallest semicircle, implying the fastest movement of charge pairs, least recombination and predicted capability of more photoactivity for g-C_3_N_4_-NS/ZnMoO_4_.The improved overall photocatalytic ability was credited to the boosted visible-radiation absorbance and low rate of charge recombination. The PC could be used for four consecutive cycles.

**Fig. 7:**
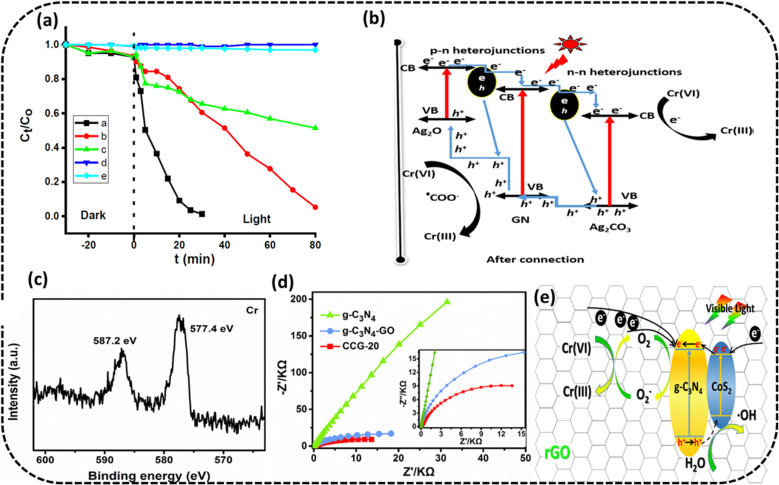
(a) Photocatalytic Cr(vi) reduction to Cr(iii) using AAG with OA, AA with OA, g-C_3_N_4_ with OA, OA and without catalyst and photolysis only (b) energy band structures of the AAG (n-Ag_2_CO_3_, p-Ag_2_O and n-g-C_3_N_4_) composite after band alignments to form p–n/n–n dual heterostructures for Cr(vi) photocatalytic reduction. Reproduced with permission from ref. 76. Copyright 2022, Elsevier (c) XPS spectra of Cr 2p on CCG-20 after reduction reaction, (d) EIS spectra of g-C_3_N_4_, g-C_3_N_4_/CoS_2_ and CCG-20 and the inset exhibits the high-resolution EIS plots for g-C_3_N_4_, g-C_3_N_4_/CoS_2_ and CCG-20 (e) schematic depicting the charge transfer process in CCG-20 and Cr(vi) photocatalytic reduction. Reproduced with permission from ref. 77. Copyright 2020, Elsevier.

Bankole *et al.* prepared Ag_2_O–Ag_2_CO_3_/g-C_3_N_4_ p–n/n–n dual heterojunctions denoted by AAG.^76^ AAG utilized the mediation of atmospheric CO_2_ and could achieve >99.5% reduction of 10 ppm Cr(vi) solution at a catalyst concentration of 0.1 g L^−1^ in the presence of oxalic acid within 30 min irradiation to visible light, as shown in Fig. 7a. Upon exposure to visible light, electrons are evicted from the V_B_ of the semiconductors (Ag included) and transferred to their respective C_B_ edges, and simultaneously, the holes with positive charges are created at the V_B_ edges. It is obvious from the band structures that the photoinduced electrons moved from high negative C_B_ edges of p-Ag_2_O, n-g-C_3_N_4_ and Ag to the least negative C_B_ of n-Ag_2_CO_3_. The highly reductive electrons accumulated on the C_B_ of n-Ag_2_CO_3_ and were captured by HCrO_4_^−1^ (at lower pH values of OA) and reduced to Cr(iii). Oxalic acid in the reaction medium used up the accumulated holes and hydroxyl radicals at the V_B_ of p-Ag_2_O to generate highly reductive anion radicals of ˙CO_2_^−^,^.^stopping the chances of the recombination of e^−^/h^+^ pairs and also reduced Cr(vi) to Cr(iii) augmenting the overall photocatalytic process, as shown in Fig. 7b. The PC could be used for five consecutive cycles.

Wang *et al.* fabricated the CoS_2_/g-C_3_N_4_ heterostructure junction backed by rGO through a one-pot solvothermal method.^77^ The improved photocatalysts could affect >99.8% removal efficiency for 20 mg L^−1^ Cr(vi) and a PC concentration of 500 mg L^−1^ under 120 min at low pH of 2 and still exhibit greater than 98% reduction efficiency, following five cycles in the same state. From the EIS plots, it is obvious that the charge transfer efficiency of the ternary nanocomposite CoS_2_/g-C_3_N_4_-rGO could be the best, indicating its excellent photocatalytic performance presented in Fig. 7c. The increased photocatalytic activity of the CoS_2_/g-C_3_N_4_-rGO ternary nanocomposite could be attributed to the increased utilization of visible light because of the coupling effect in the hybrid nanocomposite and increased SSA with additional exposed active sites of CoS_2_ scattered across rGO and the formed heterojunction of CoS_2_/g-C_3_N_4_ along with the improved separation of photogenerated charge carriers. The e^−^ and superoxide radicals (˙O_2_^−^) were probable primary dynamic species during the reduction of Cr(vi) (Fig. 7d). High-resolution XPS spectra of Cr(iii) 2p on the optimised photocatalyst surface CCG-20 after completion of the reduction reaction indicated Cr(vi) reduction to Cr(iii) (Fig. 7e).

Reddy *et al.* synthesized g-C_3_N_4_ operationalized yttrium-doped ZrO_2_ hybrid heterostructured (g-C_3_N_4_YZr) nanoparticles^78^ for photocatalytic Cr(vi) reduction. When g-C_3_N_4_ was doped with a small amount of ZrO_2_, the light adsorption ability was markedly improved because of the thin band gap. The unique arrangement of g-C_3_N_4_YZr displayed a better reduction of photocatalytic Cr(vi) because of its large surface area, lowered charge carrier recombination rate and revealed better photocatalytic activity after being exposed to sunlight for 90 minutes. The PC could withstand four repeatability tests, signifying its structural stability.

Mishra *et al.*^79^ fabricated Bi_4_O_5_I_2_/g-C_3_N_4_, p–n type direct Z-scheme heterojunction photocatalysts (BOCNs) with hierarchical 3D/2D architectures by a two-step solvothermal-calcination method. The optimised photocatalyst designated as BOCN3 exhibited Cr(vi) removal efficiency of 90.3% at a pH of 2.2 within 60 min, catalyst dose 0.4 g L^−1^ and an initial Cr(vi) concentration of 40 mg L^−1^. The photocatalyst could be tested up to the fifth successive cycle without much change in photocatalytic activity, which indicated its improved stability and reusability.

Zhao *et al.*^80^ synthesized the g-C_3_N_4_/C/Fe_2_O_3_ photocatalyst by securing g-C_3_N_4_ nanosheets onto C/Fe_2_O_3_ prepared using collagen fiber from biochar. The PC displayed improved Cr(vi) removal efficiency than pristine g-C_3_N_4_, and with the rise of Fe quantity in the g-C_3_N_4_/C/Fe_2_O_3_ photocatalyst, Cr(vi) reduction efficiency increased. The improved photocatalytic activity was credited to the indirect Z-scheme heterojunction formed amongst g-C_3_N_4_ and C/Fe_2_O_3_ that improved the separation efficiency of the light-induced charge carriers. The light-generated electrons (e^−^) were found to be the driving force for Cr(vi) removal.

Huo *et al.* fabricated the g-C_3_N_4_/BiFeO_3_/carbon nanotube ternary composite^81^ using a hydrothermal synthesis method. When the g-C_3_N_4_/BiFeO_3_/CNT composite was utilized as a PC, the photocatalytic performance improved substantially by reducing the rate of recombination of e^−^/h^+^ pairs throughout the photocatalytic reduction reaction of Cr(vi). The extra CNTs served as conduits to speed up the electron transfer procedure. Because of the high surface area, there were more active sites, which improved the photocatalytic activity. The work's findings may offer a workable foundation for treating wastewater that includes heavy metals and organic contaminants. A 93% reduction efficiency could be achieved in the case of a 5 mg L^−1^ of Cr(vi) solution in 5 h under visible irradiation.

Niu *et al.* fabricated Ag_32_NCs/g-C_3_N_4_ and Ag_9_NCs/g-C_3_N_4_ and hybrid nanocomposites^82^ by loading Ag_32_(MPG)_19_ and Ag_9_(H2MSA)_7_ nanoclusters (NCs) onto g-C_3_N_4_ utilizing a basic penetration technique to form Ag_9_-NCs/g-C_3_N_4_ and Ag_32_NCs/g-C_3_N_4_ hybrid nanocomposites. These photocatalysts could reduce 20 mg L^−1^ 100% Cr(vi) solution after 30 min and 50 min respectively under exposure to visible light. The PCs were stable enough to be recycled five times.

Chen *et al.* fabricated 3D g-C_3_N_4_@cellulose aerogels improved by cross-linked polyester fibers.^83^ Cellulose aerogels (CAs) with high permeabilities and SSA were used as a carrier for g-C_3_N_4_. As CA does not have sufficient strength and is susceptible to damage under the application of a slight force, particularly in water; therefore, the same was reinforced by blended polyester fibers (B-PET). In this case, CA with g-C_3_N_4_ nanosheet was supported by blended polyester fibers (B-PET), which increased the tensile strength of pure CA. 200 mg of PC could reduce Cr(vi) (2 × 10^−4^ mol L^−1^) to the extent of 91% in 120 min under sunlight.

### Morphological modification

The morphology of nanostructured g-C_3_N_4_ and its derivative is an important parameter from the photocatalytic standpoint. Morphology control of the g-C_3_N_4_ nanostructure appears to be a good idea as nanostructured g-C_3_N_4_ shows increased photocatalytic performance than bulk g-C_3_N_4_. Morphology alone can make a big difference to the photocatalytic activity of g-C_3_N_4_ and compounds derived from g-C_3_N_4_ as it controls the available redox reaction sites on the PC surface. g-C_3_N_4_ provides two-dimensional (2D) sheets on exfoliation, which is comprised of tri-*s*-triazine structures mutually connected *via* tertiary amines; other nanostructures like nanotubes and nanoflowers are also widely studied. Because of these types of structures, it is possible to fabricate g-C_3_N_4_-based hybrid nanocomposites by joining g-C_3_N_4_ with other components. The nanocomposites based on g-C_3_N_4_ have clear advantages over the pure g-C_3_N_4_, such as outsized surface area, more separation time, more efficiency of transportation and adequate visible light absorption. The increase in the SSA and other characteristics improves the photocatalytic ability of PC by making available more surface area to adsorb the reactants, lower charge recombination rate and the consequent higher photoreduction of Cr(vi).

**Fig. 8:**
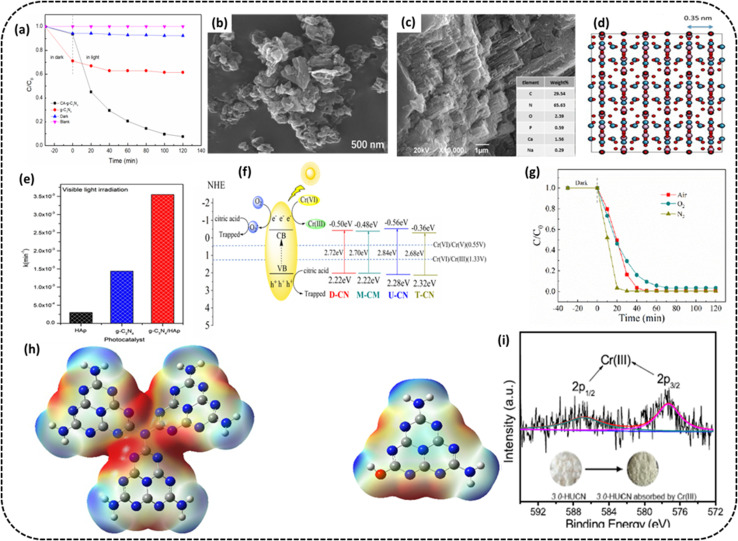
(a) Cr(vi) removal rate on g-C_3_N_4_ and CA-g-C_3_N_4_ (b) SEM image after the reaction of CA-g-C_3_N_4_. Reproduced with permission from ref. 84, Copyright 2023, MDPI (c) SEM image of the g-C_3_N_4_/HAp composite (d) structure simulation by using a zone axis [010] of hydroxyapatite Ca_5_(PO_4_)_3_(OH) (e) bar graph of reaction rate constant values for g-C_3_N_4_, hydroxyapatite, and g-C_3_N_4_/HAp composite under exposure to visible radiation. Reproduced with permission from ref. 85, Copyright 2020, Elsevier (f) Proposed mechanism for photocatalytic Cr(vi) remediation over UCN (g) rate of photocatalytic remediation of Cr(vi) by UCN. Reproduced with permission from ref. 86, Copyright 2021, MDPI (h) ESP surface distribution of optimized UCN and HUCN model (i) XPS spectrum of Cr 2p adsorbed on the 3.0-HUCN after photocatalytic reduction of Cr(vi). Reproduced with permission from ref. 87 Copyright 2020, Elsevier.

Yu *et al.* synthesized CA-g-C_3_N_4_, *i.e.*, petal-like g-C_3_N_4_ embedded with citric acid (CA).^84^ This treatment with CA changed the morphology and structure of the composite photocatalyst, enabling a more porous microstructure with a higher specific surface area and larger pore size, resulting in a rise in the number of active sites on its surface. The region of interaction between the photocatalyst and Cr(vi) was improved, causing the Cr(vi) degradation rate to 93% compared to 48% of pristine g-C_3_N_4_ under comparable circumstances (Fig. 8a). The morphology control of g-C_3_N_4_ could improve its photocatalytic activity, and the SEM image of CA-g-C_3_N_4_ is presented in Fig. 8b. The PC was stable enough to be recycled three times. The citric acid treatment effectively dispersed g-C_3_N_4_, increased the SSA of the photocatalyst, enhanced the number of active sites on the photocatalyst surface and thus enhanced the photocatalytic performance of the catalyst.

Jiménez-Flores *et al.* synthesized a g-C_3_N_4_/HAP composite by thermal condensation of melamine mixed with hydroxyapatite.^85^ As seen from the SEM image, the morphology of the g-C_3_N_4_/HAp combination exhibited a layered and piled structure common to g-C_3_N_4_ materials (Fig. 8c). The insert in the figure depicts the presence of nitrogen (65.63%), carbon (29.54%), phosphorus (0.59%), oxygen (2.39%), sodium (0.29%) and calcium (23.16%) in the g-C_3_N_4_/HAP composite. A mixture of heterogeneous phases maintaining the lamellar structure typical of pristine hydroxyapatite could be seen. Fig. 8d presents the simulated image of hydroxyapatite. The upsurge in the photocatalytic activity could be ascribed to a positive collaborative outcome created by the interaction between components, *i.e.*, g-C_3_N_4_ and hydroxyapatite impacting the surface morphology leading to improved photocatalytic behavior, as reflected in the bar chart in Fig. 8e displaying the kinetic rate constant values for g-C_3_N_4_, hydroxyapatite and g-C_3_N_4_/HAp composite under exposure to visible light. The 100% reduction of 40 ppm Cr(vi) solution was achieved after 25 and 210 min exposure in case of UV and visible light in that order. The composite was stable enough to be tested for 8 consecutive cycles.

Sun *et al.* synthesized alkali-modified g-C_3_N_4_ (*c*OH-CN) and acid-modified g-C_3_N_4_ (*c*H-CN)^88^ and reported 100% Cr(vi) could be removed in 60 min under visible radiation even though it merely demonstrated 30% in the case of the pure g-C_3_N_4_. The surface altered g-C_3_N_4_ by acid–base regulation demonstrated a larger surface area, increased pore structure abundance, a greater spectrum of visible radiation absorption, more band gap energy values, and greater ability to separate electron–hole pairs leading to efficient photocatalysis for Cr(vi) remediation.

Liang *et al.* developed a synthesis technique for g-C_3_N_4_ photocatalysts using urea, thiourea, dicyandiamide, and melamine as precursors.^86^ The varying band structure of g-C_3_N_4_ with separate morphologies derived from different precursors may be seen (Fig. 8f). The g-C_3_N_4_ derived from urea with nanosheet morphology, higher SSA, and more occupancy of surface amine groups demonstrated better than before photocatalytic activity. The morphology of the nanosheet and high surface area made it easier for charges to be separated and transmitted, which was beneficial for Cr(vi) reduction when exposed to white light irradiation. Photocatalytic remediation of Cr(vi) was made possible to the extent of 99.5% within 60 min using the urea-g-C_3_N_4_ experiment: Cr(vi) concentration at 50 mg L^−1^, urea-g-C_3_N_4_ amount = 50 mg, volume = 150 mL, citric acid = 0.9 mM at pH = 3 when exposed to white light.

Wang *et al.* fabricated an interconnected open network of hydroxyl-altered g-C_3_N_4_, *i.e.*, (HUCN)^87^ using the hydrolysis of urea-derived g-C_3_N_4_, *i.e.*, (UCN) in aqueous NaOH solution, after which self-assembly occurs *via* a dialysis process. In contrast to bulk g-C_3_N_4_, *i.e.*, (UCN), numerous exposed active sites, a quick rate of separation of photoinduced e^−^/h^+^ pairs and a greater negative conduction band edge potential are just a few of HUCN's many advantages. These benefits give the HUCN, in comparison to UCN, a noticeably better capacity for photocatalytic remediation of aqueous Cr(vi) under exposure to artificial sunlight. These observations are in line with the theoretical prediction from DFT studies. The ESP distribution obtained from the DFT calculation for the optimized models of UCN and HUCN shows a realistic pathway for electron movement at the edge of PC. As displayed in Fig. 8g and h, the blue and red colors in ESP maps denote low-electron (positive potentials) and the electron opulent (negative potentials) regions, respectively. The electron-rich areas of UCN are situated at the bridged tertiary nitrogen atoms and triangular edge nitrogen atoms in melem units. On the other hand, the electron-deprived areas remain at the other chunks of melem units (Fig. 8g). During the course of hydrolysis, the –OH groups are hosted at the HUCN edges and receive holes, sparing more electrons found at the triangular edge N atoms of melem units (Fig. 8h). It is inferred that the reduction spots have a huge shift from the internal flat part of UCN to the HUCN edges. Considering the above findings, it is concluded that progressively diminished electrochemical impedance or increased photocurrent density indicates faster photoinduced e^−^/h^+^ parting and migration capability of the HUCNs compared to UCN. This is because of the availability of –OH groups at the edges of UCNs. These –OH groups can act as h^+^ scavengers to boost the separation of the photoinduced e^−^/h^+^ pairs. Moreover, a narrow nanofiber with a width of <50 nm will be advantageous for the electrons to migrate to the surface and increase the availability of active spots. The optimized PC could reduce 99.8% Cr(vi) compared to 30.8% of the pristine g-C_3_N_4_ catalyst under identical experimental conditions, such as the PC amount of 100 mg, *C*_0_ = 20 mg L^−1^, volume = 100 mL, pH = 2.3 under artificial solar radiation. The reasons behind this exceptional photocatalytic reduction activity were as follows: The hydroxyl groups added to the HUCN's edges enhanced the contact between Cr(vi) and the HUCNs, helped to separate the charge carriers, and improved the accessibility of the active sites. Additionally, the HUCN's constructed nanostructure facilitated electron transfer to the PC surface, and the upward C_B_ edge potential gave the photoinduced electrons better reduction capability. The XPS spectrum of Cr 2p adsorbed on the 3.0-HUCN after the photocatalytic Cr(vi) remediation showed the presence of Cr(iii), implying the conversion of Cr(vi), as shown in Fig. 8i.

Wei *et al.* subjected g-C_3_N_4_ to hydrothermal treatment in aqueous HNO_3_ solutions to modify the g-C_3_N_4_ morphology.^89^ The optimised PC showed improved reduction efficiency in comparison to untreated g-C_3_N_4_ towards the photocatalytic remediation of Cr(vi). The greater photocatalytic Cr(vi) remediation activity of the hydrothermally treated g-C_3_N_4_ was mainly due to their increased SSAs, smaller particle sizes and positive surface charges, causing boosted adsorption for Cr(vi). Other reasons were the more effective separation of photoinduced e^−^/h^+^ pairs as well as the change in photocatalytic Cr(vi) reduction mechanisms. The photocatalytic Cr(vi) reduction over untreated g-C_3_N_4_ was primarily *via* a two-step ˙O_2_^−^ mediated indirect reduction mechanism. On the other hand, the same reaction over the hydrothermally treated g-C_3_N_4_ was through a one-step direct e^−^ reduction mechanism. The hydrothermal treatment appears to be a facile and useful way to enhance the Cr(vi) adsorption and photocatalytic remediation of g-C_3_N_4_.

Alam *et al.* fabricated an ACF-supported CoNiWO_4_-g-C_3_N_4_ composite.^90^ Spectroscopic analytical methods were used to validate the creation of the Z-scheme-based CNW-g-C_3_N_4_ heterostructure on the ACF substrate. Under visible light irradiation, 98.2% degradation efficiency of Cr(vi) reduction at a concentration level of 200 mg L^−1^ in 150 min, with a dose of 1 g L^−1^ CNW-g-C_3_N_4_/ACF, could be achieved. The maximum reduction rate of CNW-g-C_3_N_4_/ACF is ascribed to the shared role of adsorption and photoreduction, in which the extensive absorption of visible light and improved charge separation efficiency played a crucial role. Five repeated cycles of use showed the catalyst stability.

**Fig. 9:**
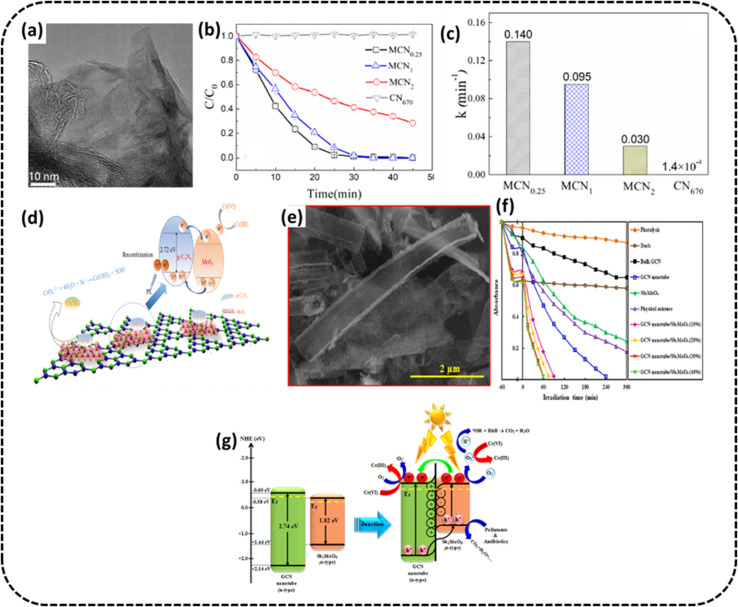
(a) TEM image of the MCN_0.25_ sample (b) variation curves of Cr(vi) concentration against time for MCN (c) photocatalytic Cr(vi) reduction rate for MCN (d) schematic of the mechanism of Cr(vi) photodegradation. Reproduced with permission from ref. 91 Copyright 2021, The American Chemical Society. (e) SEM images of the g-C_3_N_4_ nanotube/Sb_2_MoO_6_ (f) Cr(vi) removal rate over g-C_3_N_4_ nanotube/Sb_2_MoO_6_ (g) schematic for the mechanism of pollutant degradation of fabricated photocatalysts. Reproduced with permission from ref. 92, Copyright 2021, Elsevier.

Wang *et al.* constructed 2D/2D MoS_2_/g-C_3_N_4_ heterostructures^91^ for photo-remediation of Cr(vi). From the TEM image in Fig. 9a, it may be seen that small flaky pieces of MoS_2_ are evenly raised on the surface of large flaky g-C_3_N_4_. With an optimal composition denoted by MCN_0.25_, Cr(vi) could be totally reduced to Cr(iii) (100%) within 30 min under exposure to visible light at neutral pH (photocatalysts removal capacity 45 mg g^−1^), as shown in Fig. 9b. The rate constant of MoS_2_/g-C_3_N_4_ composites and pristine g-C_3_N_4_ during photoreduction of Cr(vi) are shown in the bar graph, which indicates the rate constant of MCN_0.25_ composite is about 1000 times more than pristine g-C_3_N_4_, as shown in Fig. 9c. This superior performance of PC was attributed to the chemical adsorption and photocatalytic reduction synergistically working together. Fig. 9d shows the reaction mechanism of Cr(vi) remediation from aqueous solution exposed to visible light helped by adsorption. Initially, Cr(vi), under chemisorption with MoS_2_ grids, is attached to the surface of MCN. A type II heterojunction is created between MoS_2_ with more positive conduction band potential compared to g-C_3_N_4_. The photogenerated electrons may be seen to migrate from the C_B_ of g-C_3_N_4_ to MoS_2_, and holes moving from the V_B_ of MoS_2_ to g-C_3_N_4_. The electrons on the C_B_ of MoS_2_ then attack adsorbed Cr(vi) to convert to Cr(iii) efficiently.

Hemmati-Eslamlu *et al.* synthesized n–n heterojunctions fabricated from Sb_2_MoO_6_ and g-C_3_N_4_ nanotube.^92^ The SEM image of the nanotube formation is shown in Fig. 9e. The optimised photocatalyst g-C_3_N_4_ nanotube/Sb_2_MoO_6_ (30%) sample was used for the photoreduction of Cr(vi) and the PC exhibited degradation efficiency 21.8 and 3.63-times more than that of bulk g-C_3_N_4_ and g-C_3_N_4_ nanotube samples, as shown in Fig. 9f. The probable charge migration route was suggested through the n–n heterojunction fabricated amid Sb_2_MoO_6_ and g-C_3_N_4_ nanotube, as shown in Fig. 9g. These remarkable photocatalytic improvements were credited to the boosted visible radiation absorption, fast separation of e^−^/h^+^ pairs and extended specific surface area.

Yuan *et al.* fabricated graphitic C_3_N_4_ nanosheets (g-C_3_N_4_)/ZnO amalgamated photocatalysts.^93^ The rate of photocatalytic remediation of aqueous Cr(vi) was 18% and 34% for unadulterated g-C_3_N_4_ and ZnO, respectively, under visible light for 240 min. The optimized PC could affect the photocatalytic reduction by 70% in 240 min while exposed to visible light. The PC could be recycled up to 5 times. The increased photoreduction of the g-C_3_N_4_/ZnO photocatalyst was attributed to the improved visible radiation absorption and actual split-up of photogenerated charge carriers at the interface and their transfer for the reaction.

Abdel-Moniem *et al.* synthesized nanosheets of (Bi_2_S_3_@g-C_3_N_4_) by ultrasonication.^94^ The heterostructures of Bi_2_S_3_@g-C_3_N_4_ NCs were checked for remediation of hexavalent chromium while exposed to visible light. The optimized photocatalyst Bi_2_S_3_@g-C_3_N_4_ displayed the best photoreduction activity, reaching 97% of Cr(vi) removal after 180 min under simulated solar light in the case of a 20 ppm Cr(vi) solution with 0.2 g L^−1^ of the photocatalyst. The high efficiency of the photocatalytic Cr(vi) reduction was attributed to the low combination rate of photogenerated charge carriers. The possibility of charge carrier recombination at both the surface and the bulk traps was reduced in Bi_2_S_3_@g-C_3_N_4_ nanosheets, improving the light utilization rate and increasing photocatalytic activity.

Chen *et al.* prepared porous nanosheets of g-C_3_N_4_ (PCN) with enhanced spacing^95^ between layers and more SSA using a thermal polymerization method assisted by nickel. The surface of nanosheets had more exposed active sites, and its porosity helped in the movement of photons inside the lamellar structure, and therefore, enhanced efficacy in the absorption of visible radiation. The PCN thus obtained had a higher efficiency of photocatalytic Cr(vi) reduction than pristine g-C_3_N_4_. The value of *k*, *i.e.*, the reaction rate constant of PCN (0.013 min^−1^), was nearly two times that of pristine g-C_3_N_4_ (0.007 min^−1^). The difference in the photocatalytic performance between PCN and g-C_3_N_4_ could be ascribed to higher SSA and the pores that increased absorption of visible light and a quicker path for transfer of photo-induced charges. The photoinduced e^−^was seen to be primarily responsible for Cr(vi) photocatalytic reduction. The PC could be recycled three times.

Chen *et al.* prepared ultrathin g-C_3_N_4_ nanosheets by exfoliation of raw g-C_3_N_4_ using edible glucose syrup.^96^ Compared to raw graphitic carbon nitride, the synthesized thin layers of g-C_3_N_4_ displayed an 18-fold improvement towards Cr(vi) reduction, attributable to their greater SSA and more exposed active sites. Patnaik *et al.* synthesized bimetallic alloyed Au/Pd nanoparticles embedded on nanosheets of g-C_3_N_4_ modified by mesoporous silica with a not-so-complicated one-pot calcination method.^97^ The optimized CNM-AP nanocomposite could reduce Cr(vi) C_0_ = 20 mg L^−1^, catalyst concentration = 1 g mL^−1^ to an extent of 56% under 2 h of visible light irradiation. As real-life wastewater streams contain organic materials that act as hole scavengers throughout the photocatalysis of Cr(vi) and boost the reduction reaction, the paper describes the remediation of Cr(vi) in the presence of phenol. The catalyst was found to be stable enough to be recycled four times.

### Element doping engineering

**Fig. 10:**
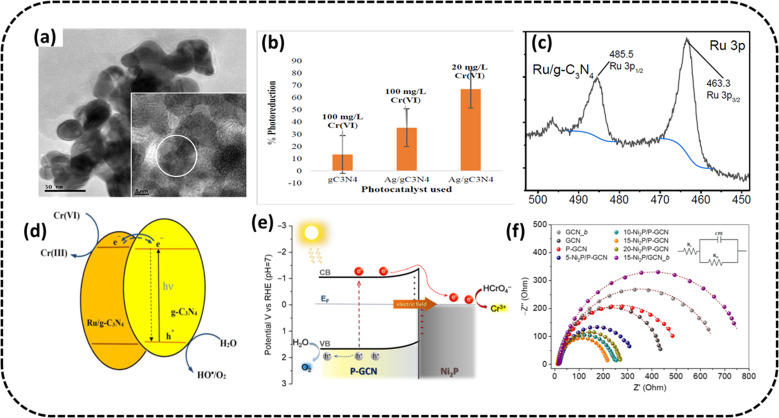
(a) TEM image (inset HRTEM image) of Ag/g-C_3_N_4_ (b) bar graph displaying Cr(vi) reduction percentage at pH 2 under irradiation from a 28 W LED light. Reproduced with permission from ref. 98, Copyright 2022, Elsevier (c) XPS spectra of the Ru 3p scan of Ru/g-C_3_N_4_ (d) schematic of the charge-migration mechanism of the photocatalytic Cr(vi) remediation using ruthenium doped g-C_3_N_4_. Reproduced with permission from ref. 100, Copyright 2023, MDPI (e) suggested mechanism for the UV-vis generated photocatalytic Cr(vi) remediation over Ni_2_P/P-g-C_3_N_4_ catalysts (f) Nyquist diagrams (inset: Randles equivalent circuit model). Reproduced with permission from ref. 99, Copyright 2023, MDPI.

Ajiboye *et al.* fabricated silver functionalized g-C_3_N_4_ by calcinating g-C_3_N_4_ with AgNO_3_.^98^ The TEM analysis displayed a flower-like morphology for the Ag-doped g-C_3_N_4_, as shown in Fig. 10a. The composite reduced 100 mg L^−1^ Cr(vi) at a low pH of value 2 to the extent of 35.3% in 120 min when exposed to visible light from a 28 W LED light, with a catalyst dosage of 50 mg. The degradation percentage was almost thrice as matched to pristine g-C_3_N_4_ (13.4%) (Fig. 10b). Under similar conditions, the PC could reduce 20 mg L^−1^ Cr(vi) to the extent of 67% in 120 min under visible light irradiation. The heightened photocatalytic activity of PC might be attributed to the decreasing rate of recombination of photogenerated e^−^/h^+^ pairs.

Xuan *et al.* prepared ruthenium-doped g-C_3_N_4_.^100^ The 5 percent Ru/g-C_3_N_4_ composite showed maximum photocatalytic activity. From the XPS analysis in Fig. 10c, the presence of Ru doping was evident with the Ru 3p_3/2_ peak values at 463.3 eV and 485.5 eV. At different concentrations of Cr(vi) (15–100 ppm) at a starting solution pH of 2.0 and catalyst concentration of 0.1 g L^−1^ with a reaction duration of 120 min, the photocatalytic efficacy of the Ru/g-C_3_N_4_ catalyst in the Cr(vi) reduction was studied. Up to 96.81% of available Cr(vi) could be reduced after 2 hours, which was twice better than the numbers for pristine g-C_3_N_4_ (50.1%). The PC could be recycled three times without loss of efficiency. The decrease in the photoinduced charge carrier recombination was identified as the cause of the increased efficacy of the Cr(vi) reduction using C_B_ electrons, which is schematically presented in Fig. 10d.

Masoumi Sangani *et al.*^101^ reported the synthesis of g-C_3_N_4_ modified with sulfanilic acid and loaded on chitosan beads (CS-GCN-S). The optimised photocatalyst of S-doped g-C_3_N_4_ nanosheets could display Cr(vi) reduction efficiency of greater than 90% within 180 min with catalyst quantity: 0.2 g L^−1^, pH: 5, Cr(vi) initial concentration: 10 mgL^−1^. The photocatalyst could be examined up to the fifth successive cycle without significant change in photocatalytic activity, implying its better stability and reusability.

Andreou *et al.* synthesized 2D/3D hybrid heterojunctions consisting of P-doped g-C_3_N_4_ nanosheets (∼50–60 nm in adjacent size) and pint-sized Ni_2_P nanoparticles of ∼5–6 nm in radius^99^ and determined their noticeable performance in the photocatalytic Cr(vi) remediation. From the EIS studies, the excellent performance of PC could be ascribed to the Ni_2_P alteration and P doping of the graphitic carbon nitride that improved the e^−^/h^+^ pair migration and spatial split-up through the boundary of Ni_2_P/P-doped g-C_3_N_4_ junctions (smallest arc diameter) (Fig. 10e). Due to these alterations, the optimised PC having 15 wt% Ni_2_P displayed better photocatalytic action in the remediation of aqueous effluents containing Cr(vi) under exposure to UV-visible radiation with 12.5% apparent quantum yield at 410 nm in the absence of sacrificial additives. PC stability was tested for three consecutive cycles. The PC could almost completely remove (>99%) (50 mg L^−1^) Cr(vi) solution in 50 min (PC concentration of 0.8 g L^−1^ at pH 1). In contrast, it took 3 h for ∼44% and ∼41% Cr(vi) remediation over g-C_3_N_4_ and P-g-C_3_N_4_ under identical conditions. The schematic in Fig. 10f shows the possible mechanism, depicting the migration of photoinduced electrons from the C_B_ of graphitic carbon nitride to Ni_2_P because of the inherent electric field created at the Ni_2_P/P-g-C_3_N_4_ Schottky junctions, where the Cr(vi) manifested as HCrO_4_^−^ was effectively converted to Cr(iii).

Jing Zhang *et al.*^102^ fabricated iron-doped graphitic carbon nitride loaded with modified dispersed diatomite. The optimal PC of 10% Fe composite with an EB dose of 30 kGy displayed improved remedial efficacy with 98.3% of available Cr(vi) in 100 min, which was nearly 16.92 times more than that of pristine g-C_3_N_4_. The PC displayed good stability even after using four times. The grand performance of PC could be attributed to Fe doping, which hindered the charge carrier recombination of photoinduced e^−^/h^+^ pairs and the capture of photoinduced carriers.

Shi *et al.*^103^ prepared a photocatalytic coating of carbon nanotubes/sulphur doped carbon nitride composite sample (CNT/SCN) by a two-step solvothermal method. The prepared CNT/SCN coating demonstrated a better photocatalytic reduction efficacy of 84.7% for Cr(vi) (10 mg L^−1^) within 8 hours under flowing water conditions. The reduction capability could surpass 75% even after three cycles of experimentation. Furthermore various other g-C_3_N_4_ modified photocatalytic systems engaged for Cr(vi) removal are summeried in table (Table 1).

**Table 1:** Summary of the numerous investigations on the removal of Cr(vi) using various g-C_3_N_4_-based nanocomposites

Sl. no.	Name of PC/nanocomposite	Reported Cr(vi) removal and PC stability. *C*_0_/ppm	References
1	g-C_3_N_4_-n–p type ZnO/BiOBr heterojunction	99%	72
		*C*_0_ = 10	
		60 min	
		Four cycles	
2	CA-g-C_3_N_4_*i.e.* petal-like g-C_3_N_4_ impregnated with citric acid (CA)	93%	84
		*C*_0_ = 20	
		Three cycles	
34	Ruthenium-modified g-C_3_N_4_	96.8%	100
		*C*_0_ = 20	
		Three cycles	
4	g-C_3_N_4_/HAP	100%	85
		*C*_0_ = 40	
		UV 25 vis 210 min	
		Eight cycles	
5	g-C_3_N_4_/Bi_2_S_3_	93.4%	73
6	Ag_3_VO_4_/g-C_3_N_4_/diatomite/DT	70%	104
		Within 60 min	
		Three cycles	
7	g-C_3_N_4_-nanosheet/ZnMoO_4_	100%	75
		*C*_0_ = 5	
		120 min	
		Four cycles	
8	g-C_3_N_4_/Ag	67%	98
		*C*_0_ = 20 mg L^−1^	
		120 min	
9	Graphitic carbon nitride functionalized with the rod-like Cu_3.21_Bi_4.79_S_9_ ternary complex	92.77%	105
		*C*_0_ = 10	
10	Ag_2_O–Ag_2_CO_3_/g-C_3_N_4_ (p–n/n–n dual heterojunctions)	89%	76
		Five cycles	
11	Ag_3_PO_4_/g-C_3_N_4_	87.6%	74
12	g-C_3_N_4_ (*c*OH-CN) and g-C_3_N_4_ (*c*H-CN)	100% in 60 min	88
13	g-C_3_N_4_/ZnIn_2_S_4_ nanocomposites	95%	106
		*C*_0_ = 100	
		60 min	
		Five cycles	
14	g-C_3_N_4_ photocatalysts using urea, thiourea. Melamine and dicyandiamide as precursors	99.5%	86
		60 min	
		UCN	
15	Bi_2_S_3_@g-C_3_N_4_ nanosheets	*C*_0_ = 10	94
		180 min	
		67.1% vis 93.1% UV	
16	Porous graphitic carbon nitride	About 91%	95
		90 min	
		Three cycles	
17	Ultrathin g-C_3_N_4_ nanosheets	18-Fold enhancement in Cr(vi) reduction	96
18	Au/Pd bimetallic alloyed nanoparticles decorated on mesoporous silica-modified g-C_3_N_4_ nanosheets	*C*_0_ = 20	97
		56% in 240 min	
		Four cycles	
19	Ag9NCs/g-C_3_N_4_ and Ag32NCs/g-C_3_N_4_ hybrid nanocomposites	*C*_0_ = 30	82
		100% in 50 min	
		Five cycles	
20	CoS_2_/g-C_3_N_4_-rGO hybrid nanocomposites	99.8% 120 min	77
		Five cycles	
21	Hydroxyl-modified graphitic carbon nitride (HUCN)	99.8% 45 min	87
		Five cycles	
22	g-C_3_N_4_/polyvinylidene fluoride composite	Alone mat 23%	107
		*C*_0_ = 30	
		240 min	
		85% with formic acid	
		Five cycles	
23	g-C_3_N_4_/C/BiFeO_3_	93%	108
24	g-C_3_N_4_ @CA/B-PET where cellulose aerogel is CA, PET stands for polyethylene terephthalate	200 mg of PC could reduce Cr(vi) (2 × 10^−4^ mol L^−1^) to the extent of 91% in 120 min under sunlight	83
25	Pd nanocones supported on g-C_3_N_4_	Up to 99.9%	109
		20 min	
		Five cycles	
26	Graphitic carbon nitride supported sulfur nanoparticles	99% in 15 min	110
		*C*_0_ = 100	
		30 mg PC	
		Seven cycles	
27	TiO_2_/g-C_3_N_4_ microspheres/reduced graphene oxide	97% after 240 min	111
		*C*_0_ = 100	
		50 mg PC	
		Five cycles	
28	Ag_3_PO_4_/g-C_3_N_4_	94.1%	31
		Five cycles	
29	ACF-supported CoNiWO_4_-g-C_3_N_4_	98.2%	90
		*C*_0_ = 200 ppm	
		150 min	
		1 g L^−1^ PC	
		Five cycles	
30	Iron-doped g-C_3_N_4_ loaded with modified dispersed diatomite	98.3% of Cr(vi) in 100 min	102
		Four cycles	
31	2D/3D Ni_2_P/P-doped g-C_3_N_4_	(>99%) (50 mg L^−1^) 50 min	99
		0.8 gL^−1^ PC at pH 1	
		Three cycles	
32	2D/2D MoS_2_/g-C_3_N_4_	(>99%)	90
		<30 min	
		PC removal capacity 45 mg g^−1^	
33	Bi_4_O_5_I_2_/g-C_3_N_4_	(90.3%)	79
		(40 mg L^−1^) 60 min	
		0.4 g L^−1^ PC at pH 2.2	
		Five cycles	
34	2D/2D MoS_2_/g-C_3_N_4_	(>90%)	101
		(10 mg L^−1^) 180 min	
		0.2 gL^−1^ PC at pH 5	
		Five cycles	
35	CNT/sulphur doped g-C_3_N_4_	(∼85%)	103
		(10 mg L^−1^) 8 hours	
		Three cycles under flowing water conditions	

## Future prospective and conclusions

Photocatalytic Cr(vi) reduction is considered an effective strategy compared to conventional methods of redemption. g-C_3_N_4_, being a non-metallic photocatalytic semiconductor material, possesses wide application prospects in the photocatalytic arena owing to non-toxicity, high photocatalytic behavior, adequate electronic structure, visible light absorption capability, abundantly available precursors and cost-effectiveness. This comprehensive review particularly focuses on the recent trend of photocatalytic Cr(vi) reduction using g-C_3_N_4_ along with the reaction mechanism. Although various modifications on g-C_3_N_4_ enhance chromium reduction remarkably, some lacunas still need to be addressed before being used in practical applications. The knowledge behind the reaction mechanism involved in photocatalytic chromium reduction is still insufficient. In order to explain the reaction mechanism taking place during the process of photocatalysis, free radical capturing experiments, RDE and ESR studies are usually preferred. The methods reported herein provide a routine understanding of the reactions and kinetics of the reactants that lack in-depth understanding. Therefore, new techniques like TPV, *in situ* XAS, and TPC can systematically elucidate the reaction procedures of Cr(vi) reduction at the molecular level.

While it is easier to augment the performance of the photocatalyst, designing active sites on it is quite tough. It is very difficult to control the atom vacancies, quantity of heteroatoms and emending active sites at the time of synthesis. Therefore, studies on developing advanced synthesis approaches should be prioritized for the preparation of g-C_3_N_4_, which can address the above-mentioned issues. The g-C_3_N_4_ production through the normal methods is still quite challenging and not recommended for various functionalizations because of the absence of abundant active sites required for surface chemical reactions. As there are a variety of strategies in the fabrication procedure of graphitic carbon nitride, with each approach altering the feature of the prepared g-C_3_N_4_, a pressing need to improvise these techniques *via* eco-friendly methods is essential. Doping Strategies like Elemental Doping that involve incorporating heteroatoms (such as N, B, S, P) into the g-C_3_N_4_ lattice can create active sites by changing the electronic structure. These dopants are capable of enhancing charge transfer and promoting specific surface reactions. Metal Doping: introducing metal atoms (*e.g.*, Co, Ni, and Pt) onto the g-C_3_N_4_ surface can create catalytically active centers. These metals can serve as electron sinks on the catalyst surface, helping charge separation and supporting redox reactions. Defect Engineering: controlled introduction of defects (such as vacancies or edge sites) can create active sites. Defects modify the nearby electronic environment, making them more favorable for adsorption followed by the reaction. Edge Sites modification: increasing the edge-to-basal plane ratio exposes more active sites. Strategies like exfoliation or edge-functionalization are capable of enhancing edge site density. Surface Functionalization: covalently attaching functional groups (*e.g.*,–NH_2_, –OH) to the g-C_3_N_4_ surface can create active sites. These groups can participate in surface reactions.

Cr(vi) reduction through three-electron oxygen reduction generally has high selectivity and the one-electron oxygen reduction path possesses a quicker rate of Cr(vi) reduction. Basically, both pathways are highly dependent on the capability of the photocatalyst to efficiently transfer e^−^ to O_2_. Introducing carbon vacancies for better absorption of O_2_ by doping alkali metals for the generation of Lewis acid sites is quite helpful. However, there is no clear evidence about which modification is more favorable for Cr(vi) reduction and which functional group is helpful for participating in the reaction. Additionally, the perfect interpretation of the mechanistic approach can only be achieved based on theoretical calculations.

One of the prerequisites for the practical application of photocatalytic Cr(vi) remediation is the long-term stability of the photocatalyst. All the studies analyzing cycling plus aging tests on photocatalysts and XRD, XPS, TEM with other characterizations for used catalysts refer to their good stability, but the test duration is comparatively short. The short-term stability of the photocatalyst may be due to its deactivation with several test runs. Therefore, it is highly essential to pump the cycling ability as long as it can be. Specially, in the case of Cr(vi) remediation, higher efficiency can be attained by varying experimental conditions such as solution pH, catalyst amount, light intensity, temperature, irradiation time, sacrificial agent amount, Cr(vi) ion concentration and photocatalyst stability.

Another concept of the surface plasmon effect in g-C_3_N_4_ can also play a necessary role in enhancing photocatalytic Cr(vi) reduction. The phenomenon of collective oscillation of free electrons upon light illumination could drastically increase the reaction processes along with better light absorption and charge separation. Hence, more studies may be conducted in this context to augment photocatalytic Cr(vi) reduction *via* g-C_3_N_4_. This includes investigation of other materials like noble metals, metal oxides with tailored LSPR properties, exploration of hybrid systems (*e.g.*, metal–semiconductor composites) to synergize LSPR with existing photocatalysts, optimization of the nanostructure design (size, shape, composition) to maximize LSPR effects, understanding the interplay between LSPR and charge carrier dynamics, studying LSPR-induced hot electrons and their role in redox reactions, and consideration of real-world applications, such as water purification or pollutant degradation.

**Fig. 11:**
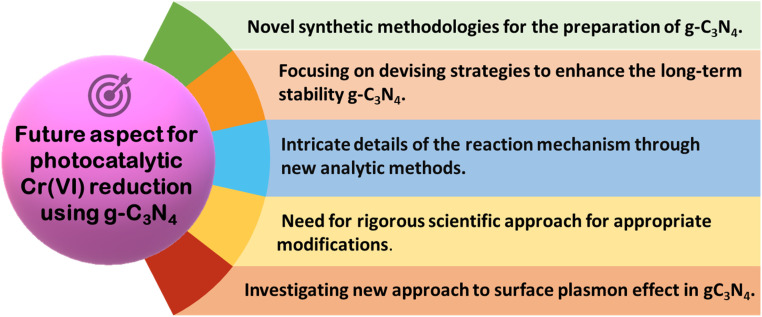
Schematic depicting the future perspective of g-C_3_N_4_-based photocatalysts towards chrome remediation.

This review is centered on work carried out so far using new-age novel graphitic carbon nitride-based nanocomposites for Cr(vi) remediation using visible light. The conversation makes it abundantly evident that the majority of these photocatalyst materials are still in the early stages of investigation in the lab. These graphitic carbon nitride-based nanocomposites are yet to be used for practical purposes. Therefore, further research is required for real-life situations using actual wastewater containing Cr(vi) in different scales and continuous operation modes. In order to advance research towards a commercial-scale ETP, more studies are required to address the large-scale synthesis of nanomaterials, develop photo reactors of the proper size, and optimize the economy for processes based on g-C_3_N_4_ nanomaterials. As real-world applications involve complex matrices, raw samples from polluted sites are required to be used in exploring field trials with g-C_3_N_4_ photocatalyst in a small solar pond. The results of the optimization study of trial runs in the pond should generate enough engineering data needed to design a pilot plant-scale reactor. Thereafter, the studies in pilot-scale plants are essential for validating catalyst performance based on which actual largescale plants may be designed and used. A summary regarding the highly required future perspective is presented in Fig. 11.

## Abbreviations

2D: Two dimensional
3D: Three dimensional
Cr(vi): Hexavalent chromium
Cr(iii): Trivalent chromium
Cr(0): Zerovalent or metallic chromium
CB: Conduction band
NPs: Nanoparticles
EA: Elemental analysis
XRD: X-ray powder diffraction
XPS: X-ray photoelectron spectroscopy
VB: Valance band
UVDRS: Ultraviolet-visible diffuse reflectance spectroscopy
PC: Photocatalyst
TEM: Transmission electron microscopy
SEM: Scanning electron microscope
PEC: Photoelectrochemical
h^+^: Holes
e^−^: Electron
–
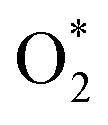
Super oxide radical
–OH*: OH radical
ROS: Reactive oxidative species
ESP: Electrostatic potential
RDE: Rotating disk electrode
ESR: Electron spin resonance
TPV: Transient photovoltaic responses
XAS: X-ray absorption spectroscopy
TPC: Transient photo current measurement
ETP: Effluent treatment plant

## Data availability

Data will be available upon reasonable request.

## Conflicts of interest

There are no conflicts to declare.
